# Studies on the Deviation of a Solution from the Hypothetical Ideal Solution with the Total Activity Coefficient

**DOI:** 10.3390/molecules30081681

**Published:** 2025-04-09

**Authors:** Yeqiu Zhou, Feiwu Chen, Yu Zhou

**Affiliations:** 1 Department of Chemistry and Chemical Engineering, School of Chemistry and Biological Engineering, University of Science and Technology Beijing, Beijing 100083, China; yeqiuzhou_bei@163.com; 2 College of Chemistry and Chemical Engineering, Qingdao University, Qingdao 266071, China

**Keywords:** activity coefficient, total activity coefficient, binary solution, non-ideal solution, intermolecular interaction energy, excess volume

## Abstract

The total activity coefficient is exploited to study the deviation of a solution from the hypothetical ideal solution. It is proven for a solution that the curve of the total activity coefficient and all curves of activity coefficients of components will intersect at the stationary point of the total activity coefficient curve. It is found for the negative (positive) deviation binary solutions studied here that the magnitudes of the total activity coefficient at the minimum (maximum) point of the total activity coefficient curve becomes bigger as the intermolecular attraction of the solute and solvent become weaker. Furthermore, the shape of the total activity coefficient curve, as well as the molar concentration of its stationary point, are dependent on the intermolecular attraction of components in the solution. Finally, for the negative solution of tetrahydrofuran + 1,1,2,2-tetrachloroethane and the positive-deviation solution of ethyl isobutyrate + 1-butanol, the effects of pressure on the total activity coefficient of the negative and positive solutions are investigated.

## 1. Introduction

The activity coefficient of component B in a solution is defined as the ratio of its activity to its concentration. It is a dimensionless, non-negative correction parameter which is used to measure the departure degree of behavior of component B from the ideal or ideally dilute behavior [1]. It plays an irreplaceable role in exploring the thermodynamic properties of solutions and metallurgical melts. When the activity coefficients of components in a solution are less than 1, this solution has a negative deviation, such as binary mixtures of trichloromethane with tetrahydropyran at 333.15 K [2] and tetrahydrofuran with 1,1,2,2-tetrachloroethane at 298.15 K [3]. When the activity coefficients of components in a solution are greater than 1, this solution has a positive deviation, such as binary mixtures of 2--methylpropene with methanol at 323.15 K [4] and ethyl isobutyrate with 1-butanol at 323.15 K [5]. When the activity coefficients of components in a solution are equal to 1, this solution is an ideal solution. The activity coefficient of a pure substance is 1.

Factors such as temperature, concentration, and components will affect the value of the activity coefficient. In terms of temperature, it has been reported that infinite dilution activity coefficients of n-hexane, n-heptane, and n-decane in polypropylene decrease with the temperature increase [6]. For metals, the activity coefficients of Dy in liquid Bi increases with increasing temperature [7]. The activity coefficients of vanadium are also positively correlated with temperature in fcc phase Pt-V alloy [8]. In terms of concentration, the average activity coefficients of many electrolytes have a non-monotonic concentration dependence. It is mainly due to the balance between solvation and ion–ion correlation terms [9,10]. In terms of components, the activity coefficients of surfactant anions are dependent on the lengths of their hydrocarbon radicals and the nature of their functional groups [11].

There are many methods to determine the activity coefficient. The electromotive force method [12,13,14,15] and cell potential method [16] use the Nernst equation to get the activity coefficient of the components. Through the Gibbs–Duhem equation, the activity coefficient of the solvent (or solute) can be determined from the activity coefficient of the solute (or solvent) [17,18]. The activity coefficient can also be determined by measuring the depression of the freezing point of the solution [19,20]. On other hand, many theoretical models have also been developed for the numerical predictions of the activity coefficient. The Wilson model [21,22,23] is derived from the concept of local composition. The NRTL model [24,25,26] is based on Scott’s two-liquid model and is applicable to partially miscible systems. The Pitzer ion interaction model [27,28,29,30] is suitable for calculating the ion activity coefficient in electrolyte solutions. The UNIQUAC model [31,32] can imitate the polymer systems with large differences in molecular size. The UNIFAC model [33,34,35] requires few parameters, including molecular structure and necessary group parameters. COSMO-RS [36,37,38] is a surface interaction model. Its parameters are established on a physical basis and depend only on the underlying quantum chemical model. In the COSMO-SAC model [39,40,41], molecules are considered to be a collection of surface segments, and activity coefficients are obtained by all contributions of these segments.

The activity coefficient can be used to calculate the excess thermodynamic properties. Excess functions include excess Gibbs free energy, excess volume, excess enthalpy, and excess entropy. Similarly to the activity coefficient, they can also measure the degree of deviation of a solution from the ideal solution. Excess properties are generally more sensitive than phase equilibria and can be used to check the accuracy of thermodynamical theories and models [42,43]. They can also be obtained through molecular simulation [44].

The total activity coefficient in our previous study [45] is used to investigate the deviation of solution from the hypothetical ideal solution. Two negative-deviation solutions and four positive-deviation solutions are chosen for the present study. Variations in the total activity coefficient with the molar concentration in negative and positive solutions are investigated from the viewpoint of the intermolecular interaction. The effect of pressure on the total activity coefficient is also studied for the negative and positive-deviation solutions.

## 2. Results and Discussion

Two negative-deviation systems and four positive-deviation systems are chosen for this study. The negative-deviation systems are trichloromethane + tetrahydropyran and trichloromethane + piperidine [2]. The positive-deviation systems are 2-methylpropene + methanol, 2-methylpropene + ethanol, 2-methylpropene + 2-propanol, and 2-methylpropene + 2-butanol [4].

The deviation of a solution from the hypothetical ideal solution can be interpreted from the viewpoint of the intermolecular interaction [1]. For a binary solution, the two components are denoted separately as A and B. If the intermolecular attraction of A···B is stronger than the average of the A···A and B···B attractions, the binary solution has a negative deviation; If the intermolecular attraction of A···B is weaker than the average of the A···A and B···B attractions, the solution has a positive deviation. In this study, the interactions between the components of a solution are calculated by the Gaussian 09 program [46].

The geometries of components A and B of a solution of interest are optimized by the Gaussian 09 program at the B3LYP-D3(BJ)/6-311+G* level. Using the optimized structures of A and B, fifty different initial geometrical guesses for the complexes A···A, B···B, and A···B are generated by the Molclus program [47], respectively. MOPAC [48] is employed to pre-optimize all these initial geometrical guesses. Then, the five structures with the lowest energies are selected from the fifty pre-optimized structure set and are further optimized with B3LYP-D3(BJ)/6-311+G* by the Gaussian 09 program. The structure with the lowest energy is finally chosen to calculate the interaction energies of the corresponding complex pairs, A···A, B···B, and A···B, using the Gaussian 09 program with B3LYP-D3(BJ)/6-311+G*. The interaction energies are estimated as the energy difference between each complex and the sum of the isolated monomers, and are further corrected with the basis set superposition error (BSSE) using the counterpoise method proposed by Boys and Bernardi [49].

In addition, the Interaction Region Indicator (IRI) [50] is exploited to reveal both chemical bonds and weak interactions in chemical systems. IRI analysis is performed on the most stable structures of A···A, B···B, and A···B complexes. IRI is calculated using the wave function analysis software Multiwfn [51]. The results are visualized by VMD 1.9.3 [52].

### 2.1. Negative-Deviation Solutions

**Figure 1:**
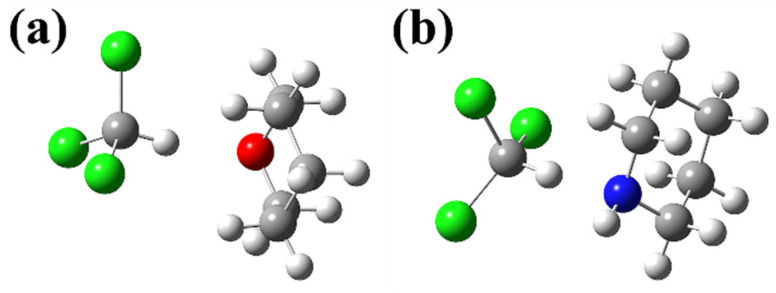
Optimized geometries of (**a**) trichloromethane + tetrahydropyran and (**b**) trichloromethane + piperidine complexes. The white balls are H atoms, the gray balls are C atoms, the green balls are Cl atoms, the red ball is an O atom, and the blue ball is an N atom.

Two negative-deviation solutions are, separately, trichloromethane (CHCl_3_) + tetrahydropyran (*c*-(CH_2_)_5_O) and trichloromethane + piperidine (*c*-(CH_2_)_5_NH). The ball-and-stick models of CHCl_3_···*c*-(CH_2_)_5_O and CHCl_3_···*c*-(CH_2_)_5_NH complexes are presented in Figure 1. These structures are the most stable structures.

**Figure 2:**
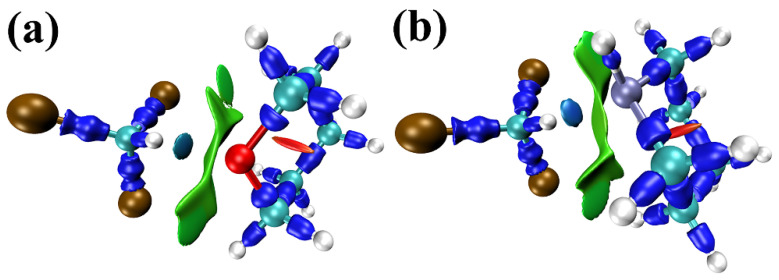
IRI isosurface maps of (**a**) trichloromethane + tetrahydropyran and (**b**) trichloromethane + piperidine. The white balls are H atoms, the greenish-blue balls are C atoms, the brown balls are Cl atoms, the red ball is an O atom, and the light purple ball is an N atom.

The IRI isosurface maps of the two complexes above are demonstrated in Figure 2. All kinds of interactions are clearly revealed in these maps. Figure 2a shows the interaction between trichloromethane and tetrahydropyran molecules. The dark blue isosurfaces correspond to chemical bonds. The blue isosurface between the two monomers vividly represents a significant attraction between the H atom of the trichloromethane molecule and the O atom of the tetrahydropyran molecule. In Figure 1a, the distance between these two atoms is 2.01 Å. The sum of the van der Waals atomic radii for H and O is 2.5 Å [53]. As the existence of hydrogen bonds can be judged by the sum of the van der Waals atomic radii [54], this blue isosurface is proven to represent a hydrogen bond. The green isosurface indicates obvious van der Waals interactions between trichloromethane and tetrahydropyran molecules. The red isosurface highlights the strong steric effect in the small ring of the tetrahydropyran molecule. But this is an intramolecular interaction, which will not be discussed in this study. All of these illustrate that the interaction between two molecules is mutual attractive. Figure 2b shows the interaction between trichloromethane and piperidine molecules. Similarly, there is also a blue isosurface that implies a hydrogen bond between the H atom of the trichloromethane molecule and the N atom of the piperidine molecule. This is more evidence to prove the existence of this hydrogen bond: The sum of the van der Waals atomic radii for H and N is 2.6 Å [53], which is greater than the 2.04 Å distance between the H atom of the trichloromethane molecule and the N atom of the piperidine molecule in Figure 1b. The color of the blue isosurface in Figure 2b is darker than the color of blue isosurface in Figure 2a. This means that the strength of this C-H···N hydrogen bond is stronger than the strength of this C-H···O hydrogen bond. The larger green isosurface in Figure 2b indicates more van der Waals interactions between the trichloromethane molecule and the piperidine molecule. These means that the total interaction of the CHCl_3_···*c*-(CH_2_)_5_NH complex stronger than the total interaction of the CHCl_3_···*c*-(CH_2_)_5_O complex.

**Figure 3:**
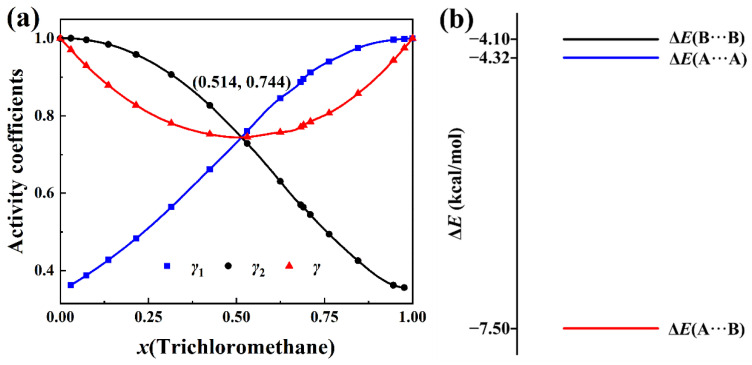
(**a**) Plots of *γ*(total activity coefficient), *γ*_1_(trichloromethane), and *γ*_2_(tetrahydropyran) versus *x*(trichloromethane) at 333.15 K. (**b**) BSSE-corrected intermolecular interaction energies of the three complexes in the trichloromethane (A) + tetrahydropyran (B) solution calculated at the B3LYP-D3(BJ)/6-311+G* level.

Based on the activity coefficients of the components of the trichloromethane (CHCl_3_) + tetrahydropyran (*c*-(CH_2_)_5_O) solution at 333.15 K and the corresponding molar concentration of trichloromethane in the literature [2], the total activity coefficient is calculated by Equation (8) in Section 3. The results can be seen in Table S1 (Supplementary Materials). Figure 3a depicts the total activity coefficient and activity coefficients of the two components as the functions of the molar concentration of trichloromethane. The curves are drawn by cubic spline fitting. The coordinates of the minimum point of the curve of the total activity coefficient are marked in the graph.

In Figure 3a, as the molar concentration of trichloromethane increases, the activity coefficient (*γ*_1_) of trichloromethane increases monotonically, while the activity coefficient (*γ*_2_) of tetrahydropyran decreases monotonically. Because of the opposite variation trends of *γ*_1_ and *γ*_2_ with *x*(trichloromethane), it is difficult to determine the degree of non-ideality of the solution just from the magnitudes of *γ*_1_ and *γ*_2_. On the other hand, there is no such problem for the total activity coefficient. The total activity coefficient of the solution decreases first and then increases with the increase in the molar concentration of trichloromethane. The largest deviation (*γ* = 0.744) from the hypothetical ideal solution is at *x*(trichloromethane) = 0.514 at the minimum point of total activity coefficient *γ* curve. It is demonstrated in Figure 3a that the curves of *γ*_1_, *γ*_2_, and *γ* all intersect at *x*(trichloromethane) = 0.514, which corresponds to a minimum point of total activity coefficient *γ* curve. At this point, *γ*_1_ = *γ*_2_ = *γ* = 0.744. These are the confirmations of the proof presented in Section 3.

It can also be seen in Figure 3a that the total activity coefficients and the activity coefficients of trichloromethane and tetrahydropyran are all smaller than 1, which indicates that this solution has a negative deviation from the hypothetical ideal solution. The interaction energies (Δ*E*) of three types of complexes of the CHCl_3_ + *c*-(CH_2_)_5_O system with BSSE correction at the B3LYP-D3(BJ)/6-311+G* level are depicted in Figure 3b. The order of the three energies in Figure 3b is Δ*E*(*c*-(CH_2_)_5_O···*c*-(CH_2_)_5_O) > Δ*E*(CHCl_3_···CHCl_3_) > Δ*E*(CHCl_3_···*c*-(CH_2_)_5_O). Δ*E*(CHCl_3_···*c*-(CH_2_)_5_O) is well below the average of Δ*E*(*c*-(CH_2_)_5_O···*c*-(CH_2_)_5_O) and Δ*E*(CHCl_3_···CHCl_3_). This implies that trichloromethane and tetrahydropyran molecules in solution have an affinity for each other and are reluctant to escape each other’s close company by vaporizing, which leads to a negative deviation from the hypothetical ideal solution.

**Figure 4:**
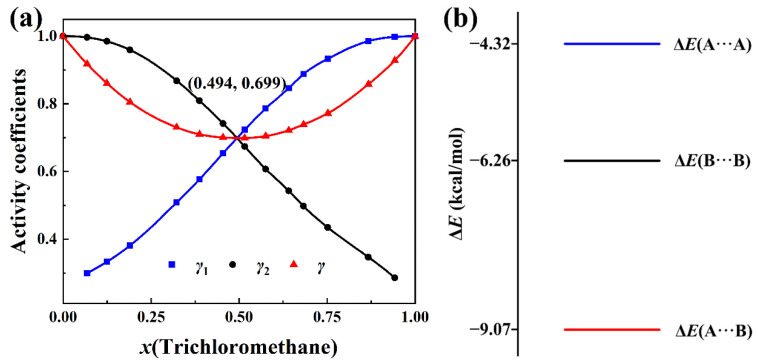
(**a**) Plots of *γ*(total activity coefficient), *γ*_1_(trichloromethane), and *γ*_2_(piperidine) versus *x*(trichloromethane) at 333.15 K. (**b**) BSSE-corrected intermolecular interaction energies of the three complexes in the trichloromethane (A) + piperidine (B) solution calculated at the B3LYP-D3(BJ)/6-311+G* level.

The data of the activity coefficients of the components of the trichloromethane + piperidine solution with a molar concentration of trichloromethane at 333.15 K are also available in the same literature [2]. The total activity coefficients calculated with Equation (8), together with activity coefficient of components of the solution, are plotted in Figure 4a. The total activity coefficient data can be found in Table S1 (Supplementary Materials). The shape of the curves in Figure 4a is similar to the shape of the curves in Figure 3a. As expected, the three curves intersect at the minimum point of the curve of the total activity coefficient *γ* in Figure 4a.

The intermolecular interaction energies of three pairs in the mixture of CHCl_3_ (A) + *c*-(CH_2_)_5_NH (B) liquids are presented in Figure 4b. Similarly to the trichloromethane + tetrahydropyran solution, Δ*E*(CHCl_3_···*c*-(CH_2_)_5_NH) in Figure 4b is much smaller than Δ*E*(CHCl_3_···CHCl_3_) and Δ*E*(*c*-(CH_2_)_5_NH···*c*-(CH_2_)_5_NH). Comparison of Figure 4b with Figure 3b shows that Δ*E*(CHCl_3_···*c*-(CH_2_)_5_O) > Δ*E*(CHCl_3_···*c*-(CH_2_)_5_NH). As discussed in Figure 2, this corresponds to the stronger intermolecular hydrogen bonds and intermolecular van der Waals interactions in the CHCl_3_···*c*-(CH_2_)_5_NH solution than in the CHCl_3_···*c*-(CH_2_)_5_O solution. Thus, the CHCl_3_ + *c*-(CH_2_)_5_NH solution deviates much more negatively from the hypothetical ideal solution than the CHCl_3_ + *c*-(CH_2_)_5_O solution. This explains why the value (0.699) of *γ* at the minimum point of the total activity coefficient curve in Figure 4a is smaller than the value (0.744) of *γ* at the minimum point of the total activity coefficient curve in Figure 3a.

On the other hand, comparing Figure 4a with Figure 3a, it is also observed that the value of the molar concentration of CHCl_3_, *x*(CHCl_3_), is related to intermolecular interactions of pure components in the solution. In Figure 3b, Δ*E*(CHCl_3_···CHCl_3_) < Δ*E*(*c*-(CH_2_)_5_O···*c*-(CH_2_)_5_O), *x*(CHCl_3_) = 0.514. But Δ*E*(CHCl_3_···CHCl_3_) > Δ*E*(*c*-(CH_2_)_5_NH···*c*-(CH_2_)_5_NH) in Figure 4b, *x*(CHCl_3_) = 0.494. These indicate, for the negative-deviation solution, that the component with a stronger (weaker) intermolecular interaction will have a molar concentration bigger (smaller) than 0.5 at the minimum point of the curve of the total activity coefficient. Similar phenomena are also presented in positive-deviation solutions.

### 2.2. Positive-Deviation Solutions

**Figure 5:**
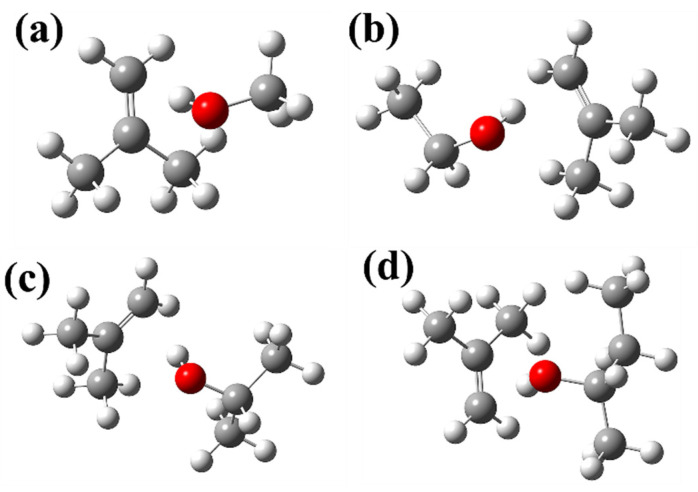
Optimized geometries of (**a**) 2-methylpropene + methanol, (**b**) 2-methylpropene + ethanol, (**c**) 2-methylpropene + 2-propanol, and (**d**) 2-methylpropene + 2-butanol complexes. The white balls are H atoms, the gray balls are C atoms, and the red balls are O atoms.

There are four positive-deviation solutions, 2-methylpropene ((CH_3_)_2_C=CH_2_) + methanol (CH_3_OH), 2-methylpropene + ethanol (CH_3_CH_2_OH), 2-methylpropene + 2-propanol ((CH_3_)_2_CHOH), and 2-methylpropene + 2-butanol (CH_3_CH_2_CH(OH)CH_3_). Figure 5 shows the ball-and-stick models of the most stable structures of these complexes. It can be seen that the O atoms of the four alcohols in Figure 5a–d are all close to the C=C bond of their corresponding 2-methylpropylene molecules. These areas may have weak interactions.

**Figure 6:**
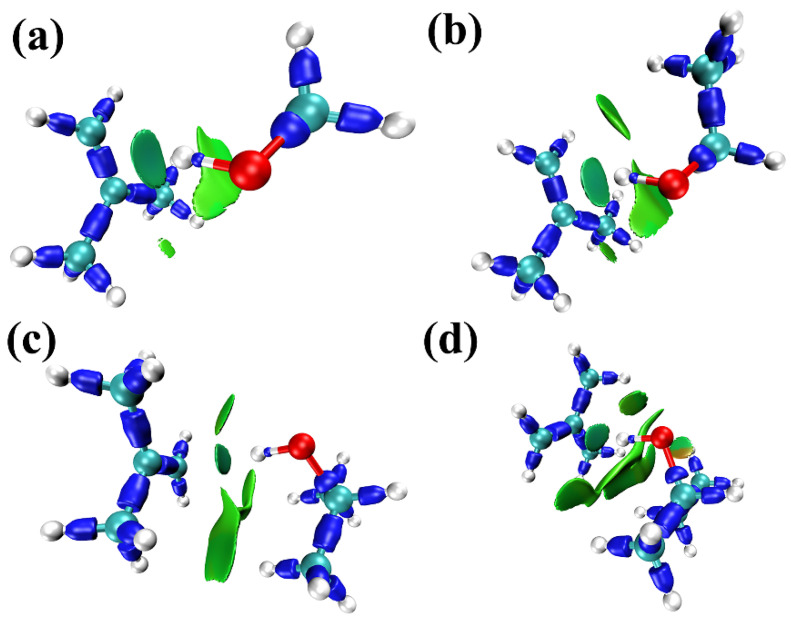
IRI isosurface maps of (**a**) 2-methylpropene + methanol, (**b**) 2-methylpropene + ethanol, (**c**) 2-methylpropene + 2-Propanol, (**d**) 2-methylpropene +2-butanol. The white balls are H atoms, the greenish-blue balls are C atoms, and the red balls are O atoms.

IRI isosurfaces of the complexes in Figure 6a–d demonstrate the intermolecular interactions between 2-methylpropene, methanol, ethanol, 2-propanol, and 2-butanol, respectively. The dark blue isosurfaces correspond to chemical bonds. The disperse green isosurface indicates significant van der Waals interactions between two molecules. There is also a small part of the isosurface exhibiting a marginally red color, implying that there may exist weak steric repulsion. However, it is an intramolecular interaction and will not be discussed further here. Based on the differences observed in these IRI isosurface maps, the order of the strengths of interaction for these complexes is likely to be

(CH_3_)_2_C=CH_2_···CH_3_OH < (CH_3_)_2_C=CH_2_···CH_3_CH_2_OH <

(CH_3_)_2_C=CH_2_···(CH_3_)_2_CHOH < (CH_3_)_2_C=CH_2_···CH_3_CH_2_CH(OH)CH_3_.

**Figure 7:**
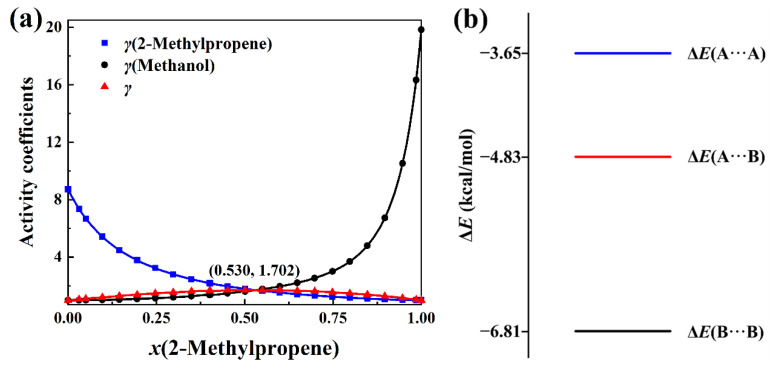
(**a**) Plots of *γ*(total activity coefficient), *γ*(2-methylpropene), and *γ*(methanol) versus *x*(2-methylpropene) at 323.15 K. (**b**) BSSE-corrected interaction energies of three complexes in the (CH_3_)_2_C=CH_2_(A) + CH_3_OH(B) system at the B3LYP-D3(BJ)/6-311+G* level.

**Figure 8:**
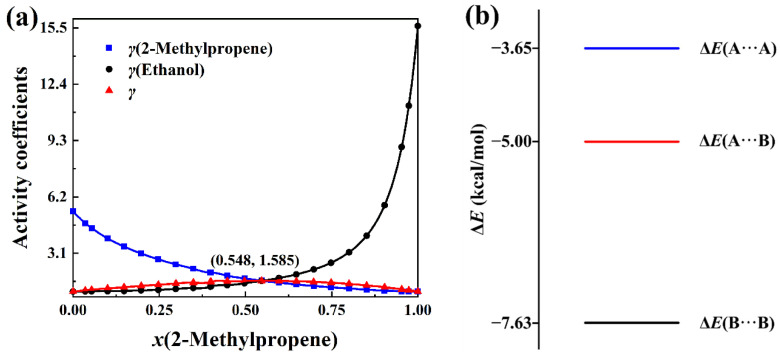
(**a**) Plots of *γ*(total activity coefficient), *γ*(2-methylpropene), and *γ*(ethanol) versus *x*(2-methylpropene) at 323.15 K. (**b**) BSSE-corrected interaction energies of three complexes in the (CH_3_)_2_C=CH_2_(A) + CH_3_CH_2_OH(B) system at the B3LYP-D3(BJ)/6-311+G* level.

**Figure 9:**
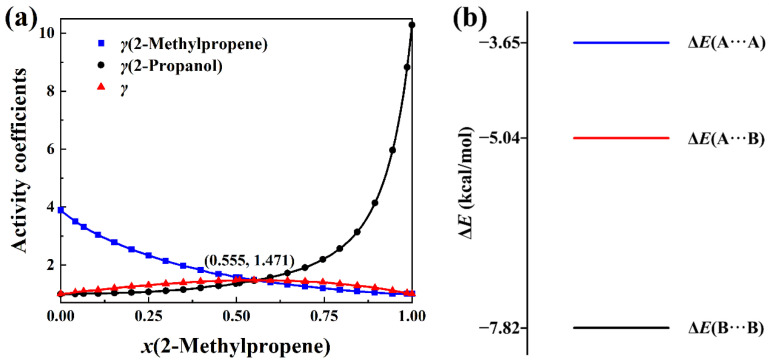
(**a**) Plots of *γ*(total activity coefficient), *γ*(2-methylpropene), and *γ*(2-propanol) versus *x*(2-methylpropene) at 323.15 K. (**b**) BSSE corrected interaction energies of three complexes in the (CH_3_)_2_C=CH_2_(A) + (CH_3_)_2_CHOH(B) system at the B3LYP-D3(BJ)/6-311+G* level.

**Figure 10:**
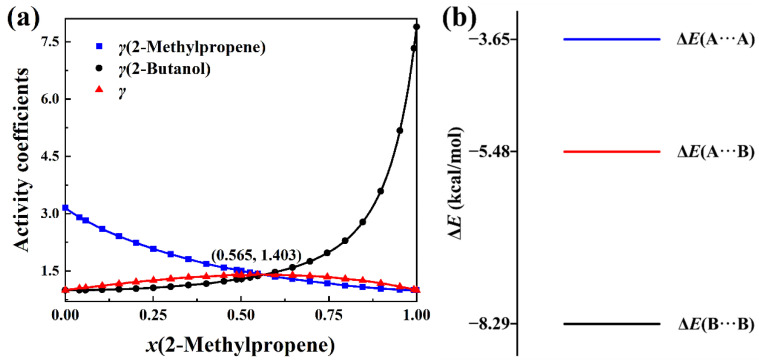
(**a**) Plots of *γ*(total activity coefficient), *γ*(2-methylpropene), and *γ*(2-butanol) versus *x*(2-methylpropene) at 323.15 K. (**b**) BSSE-corrected interaction energies of three complexes in the (CH_3_)_2_C=CH_2_(A) + CH_3_CH_2_CH(OH)CH_3_(B) system at the B3LYP-D3(BJ)/6-311+G* level.

The total activity coefficients of 2-methylpropene + methanol, 2-methylpropene + ethanol, 2-methylpropene + 2-propanol, and 2-methylpropene + 2-butanol solutions are calculated with Equation (8) based on the experimental activity coefficients of components and molar concentrations in the literature [4]. The measurement was conducted at 323.15 K. The total activity coefficient data can be seen in Tables S2 and S3 (Supplementary Materials). The plots of the total activity coefficient and activity coefficients of components versus the molar concentrations, as well as the intermolecular interaction energies, are presented in Figure 7, Figure 8, Figure 9 and Figure 10. In opposition to the concave profiles of the total activity coefficient curves in Figure 3a and Figure 4a, the total activity coefficient curves of four positive-deviation solutions in Figure 7a, Figure 8a, Figure 9a and Figure 10a have convex shapes.

It can be seen from Figure 7a, Figure 8a, Figure 9a and Figure 10a that the total activity coefficient curve and the two activity coefficient curves of the components all intersect at the maximum point of the total activity coefficient curves, as proved in Section 3. In the negative-deviation solutions shown in Figure 3a and Figure 4a, the activity coefficients of the components are in the interval from 0 to 1. However, the activity coefficients of the components in Figure 7a, Figure 8a, Figure 9a and Figure 10a are all much bigger than 1. The activity coefficient 19.82 of methanol is the biggest among the four solutions. The total activity coefficients of the four solutions are in the range 1 to 2, which indicates that these mixtures of liquids are positive-deviation solutions.

The intermolecular interaction energies of 2-methylpropene + methanol, 2-methylpropene + ethanol, 2-methylpropene + 2-propanol, and 2-methylpropene + 2-butanol solutions are presented in Figure 7b, Figure 8b, Figure 9b and Figure 10b, respectively. The interaction energies of the A···B pairs in these figures are all above the average interaction energies of the A···A and B···B pairs. Because of this, the components of these solutions feel less attractive toward each other and have a larger tendency to escape each other’s company by vaporizing. Therefore, the four solutions are thus positively deviated from the hypothetical ideal solution.

As for the negative-deviation solutions in Figure 3b and Figure 4b, the magnitude of the total activity coefficient at the minimum point of the total activity coefficient curve becomes smaller as the intermolecular attraction of A and B becomes stronger. Similarly, the magnitude of the total activity coefficient at the maximum point of the total activity coefficient curve in Figure 7a, Figure 8a, Figure 9a and Figure 10a becomes bigger as the corresponding intermolecular attraction of A and B becomes weaker. On the other hand, the molar concentration *x*_A_ of the maximum point of the total activity coefficient curve becomes bigger than 0.5 as the intermolecular attraction of B and B becomes stronger, i.e., the maximum points of the total activity coefficient curves move horizontally to the side of the pure component with weak intermolecular attraction. In opposition to the positive-deviation solutions, the minimum points of the total activity coefficient curves in Figure 3a and Figure 4a move horizontally to the side of the pure component with strong intermolecular attraction, as discussed at the beginning of this Section.

### 2.3. Partial Derivative of the Total Activity Coefficient

In order to determine the partial derivative of the natural logarithm of the total activity coefficient with respect to pressure at constant temperature, there are two terms in Equation (20) in Section 3, ∂*x*_B_/∂*P* and ln(*γ*_B_/*γ*_A_), needed to be calculated. Two solutions with separately negative and positive deviations from the hypothetical ideal solution are chosen for this study. The negative- and positive-deviation solutions are tetrahydrofuran + 1,1,2,2-tetrachloroethane [3] and ethyl isobutyrate + 1-butanol [5], respectively.

**Figure 11:**
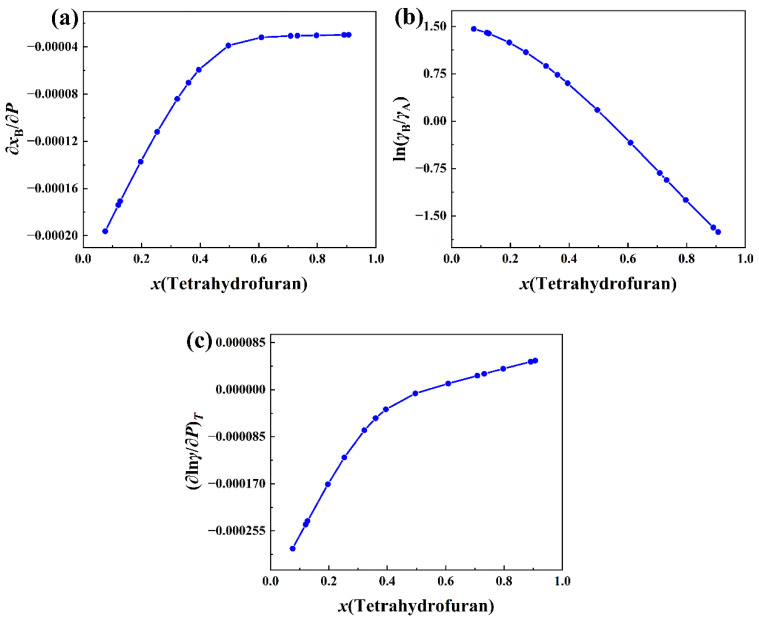
(**a**) Plot of ∂*x*_B_/∂*P* versus *x*(tetrahydrofuran) in tetrahydrofuran(A) + 1,1,2,2-tetrachloroethane(B) system at 298.15 K. (**b**) Plot of ln(*γ*_B_/*γ*_A_) versus *x*(tetrahydrofuran) in this system at 298.15 K. (**c**) Plot of (∂ln*γ*/∂*P*)*_T_* versus *x*(tetrahydrofuran) in this system at 298.15 K.

Because the experimental data of $V_{m}^{E}$ and pressure are measured at different molar concentrations of 1,1,2,2-tetrachloroethane in tetrahydrofuran (A) + 1,1,2,2-tetrachloroethane (B) solution at 298.15, *x*_B_ is fitted as a function of *P* as described below:

(1) $$x_{B}=a+b_{1}(1-e^{-\frac{P}{c_{1}}})+b_{2}(1-e^{-\frac{P}{c_{2}}})$$

where *a*, *b*_1_, *c*_1_, *b*_2_, and *c*_2_ are fitting parameters. *x*_B_ is the molar concentration of 1,1,2,2-tetrachloroethane. The range of variation for *P* is 889 Pa to 20,304 Pa. The molar concentration *x*_A_ of tetrahydrofuran is simply 1 − *x*_B_. The experimental data [3] of *x*_B_ and pressure *P* are fitted with Equation (1) and are shown in Figure 11a. The experimental data of *γ*_A_ and *γ*_B_ are also available in the literature [3]. ln(*γ*_B_/*γ*_A_) is fitted by the cubic spline function of *x*_B_. The curve of ln(*γ*_B_/*γ*_A_) versus the molar concentration of tetrahydrofuran is plotted in Figure 11b. Once ∂*x*_B_/∂*P* and ln(*γ*_B_/*γ*_A_) are available, (∂ln*γ*/∂*P*)*_T_* can be calculated by Equation (20) together with the experimental data of $V_{m}^{E}$ [3], and these are presented in Figure 11c.

In Figure 11a, ∂*x*_B_/∂*P* is a monotonically increasing function of the molar concentration of tetrahydrofuran. It is almost negative for the entire range of the molar concentration of tetrahydrofuran and gradually approaches zero from below for relatively large molar concentrations of tetrahydrofuran. In Figure 11b, ln(*γ*_B_/*γ*_A_) is a monotonically decreasing function. As can be seen from Figure 11c, the partial derivative (∂ln*γ*/∂*P*)*_T_* is an increasing function of the molar concentration of tetrahydrofuran and changes from negative to positive. It implies that the total activity coefficient will decrease with the increase in pressure for smaller molar concentrations of tetrahydrofuran, and increase with the pressure for relatively large molar concentrations of tetrahydrofuran. The molar concentration of the dividing point is around 0.5. Since the contribution of the term involving $V_{m}^{E}$ to (∂ln*γ*/∂*P*)*_T_* is much smaller than the contribution of the second term on the right side of Equation (20), the molar concentration of the dividing point in Figure 11c is, in fact, the same as the molar concentration of the zero point of ln(*γ*_B_/*γ*_A_), as can be seen from Figure 11b.

**Figure 12:**
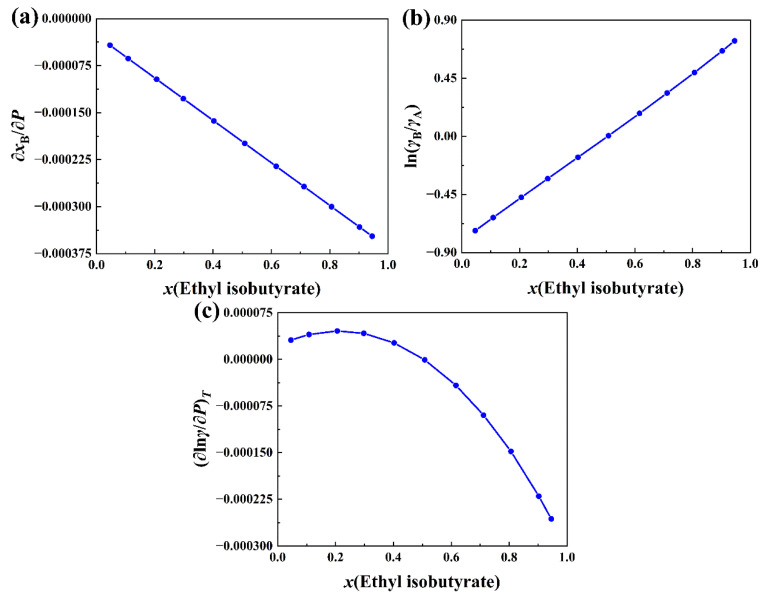
(**a**) Plot of ∂*x*_B_/∂*P* versus *x*(ethyl isobutyrate) in ethyl isobutyrate(A) + 1-butanol(B) system at 323.15 K. (**b**) Plot of ln(*γ*_B_/*γ*_A_) versus *x*(ethyl isobutyrate) in this system at 323.15 K. (**c**) Plot of (∂ln*γ*/∂*P*)*_T_* versus *x*(ethyl isobutyrate) in this system at 323.15 K.

Like the negative-deviation solution of tetrahydrofuran + 1,1,2,2-tetrachloroethane, *x*_B_ of the positive-deviation solution of ethyl isobutyrate (A) + 1-butanol (B) should also be fitted as a function of *P*. It is found that the following function has the best performance:

(2) $$x_{B}=a+be^{cP}$$

where *a*, *b*, and *c* are fitting parameters. *x*_B_ is the molar concentration of 1-butanol. The range of variation for *P* is 4505 Pa to 11,660 Pa. The molar concentration *x*_A_ of ethyl isobutyrate is simply 1 − *x*_B_. The plot of ∂*x*_B_/∂*P* versus molar concentration of ethyl isobutyrate is shown in Figure 12a. ∂*x*_B_/∂*P* is a linearly decreasing function of the molar concentration of ethyl isobutyrate. Like *x*_B_ and *P*, the experimental data of *γ*_A_ and *γ*_B_ at 323.15 K are also available in the literature [5]. ln(*γ*_B_/*γ*_A_) is fitted by the cubic spline function of *x*_B_. The plot of ln(*γ*_B_/*γ*_A_) versus molar concentration of ethyl isobutyrate is presented in Figure 12b. However, n(*γ*_B_/*γ*_A_) is a linearly increasing function. Comparing Figure 12a,b with Figure 11a,b, it is found that the variation trends of the former are opposite to the variation trends of the latter. With the excess volume at the molar concentration of ethyl isobutyrate [5], (∂ln*γ*/∂*P*)*_T_* is calculated with Equation (20). The results are plotted against the molar concentration of ethyl isobutyrate and are presented in Figure 12c. (∂ln*γ*/∂*P*)*_T_* in Figure 12c increases with a small molar concentration of ethyl isobutyrate at first, and then decreases after passing the maximum point of the plot. The molar concentration of the maximum point is about 0.22. Because of very small magnitude of $V_{m}^{E}$, (∂ln*γ*/∂*P*)*_T_* depends mainly on the product of ∂*x*_B_/∂*P* and ln(*γ*_B_/*γ*_A_). Since ∂*x*_B_/∂*P* and ln(*γ*_B_/*γ*_A_) are linear functions of the molar concentration of ethyl isobutyrate, it is not surprising that their product (∂ln*γ*/∂*P*)*_T_* is a quadratic polynomial of the molar concentration with a maximum stationary point. In opposition to the variation of the total activity coefficient of the negative-deviation solution discussed above, the total activity coefficient in Figure 12c increases with the pressure for small molar concentrations of ethyl isobutyrate at first and then decreases with the increase of pressure for big molar concentrations. The molar concentration of the dividing point is about 0.50, which is the same as the as the molar concentration of the zero point of ln(*γ*_B_/*γ*_A_), as can be seen from Figure 12b. As for both negative and positive-deviation solutions, the molar concentration of the zero point of ln(*γ*_B_/*γ*_A_) is, in fact, equal to the molar concentration of the intersection point of the curves of *γ*, *γ*_A_, and *γ*_B_.

## 3. Materials and Methods

### 3.1. Total Activity Coefficient

The total activity coefficient theory is introduced briefly here. Under the condition of constant temperature and pressure, the chemical potential of component B in a non-ideal solution is expressed as [1]

(3) $$\mu_{B}\left(T,P\right)=\mu_{B}^{*}\left(T,P\right)+RT\ln a_{B}$$

where $\mu_{B}^{*}$, *T*, *P*, *R*, and *a*_B_ are the chemical potential of pure liquid B, the system’s temperature and pressure, the gas constant, and the activity of component B, respectively. *a*_B_ = *x*_B_*γ*_B_, where *x*_B_ and *γ*_B_ are, separately, the molar concentration and activity coefficient of component B. The partial derivative of Equation (3) with respect to *P* at constant *T* can be expressed as [45]

(4) $$V_{B}=V_{B}^{*}+RT{\left(\frac{\partial \ln\gamma_{B}}{\partial P}\right)}_{T}$$

where *V*_B_ is the partial molar volume of component B and $V_{B}^{*}$ is the molar volume of pure liquid B. According to [45], the total activity coefficient *γ* of the non-ideal solution is defined as

(5) $$V_{m}\left(\mathrm{aq}\right)=V_{m}\left(\mathrm{id}\right)+RT{\left(\frac{\partial \ln\gamma }{\partial P}\right)}_{T}$$

where *V*_m_(aq) and *V*_m_(id) represent the molar volume of actual and the hypothetical ideal solutions, respectively. The expression of *V*_m_(id) is [45]

(6) $$V_{m}\left(\mathrm{id}\right)=\frac{1}{n}\underset{B}{\sum }n_{B}V_{B}^{*}$$

where *n* is the total number of moles of the solution and *n*_B_ is the number of moles of component B. The total volume of the solution, *V*, can be expressed as [45]

(7) $$nV_{m}\left(\mathrm{aq}\right)=V=\underset{B}{\sum }n_{B}V_{B}$$

Substituting Equation (5) and Equation (4) into the left- and right-hand sides of Equation (7), after some arrangements, Equation (7) becomes *n*(∂ln*γ*/∂*P*)*_T_* = $\underset{B}{\sum }n_{B}$(∂ln*γ*_B_/∂*P*)*_T_*. Its integration form is *γ^n^ =* $\underset{B}{\prod }\gamma_{B}^{n_{B}}$. Taking the natural logarithm of this equation leads to [45]

(8) $$\ln\gamma =\underset{B}{\sum }x_{B}\ln\gamma_{B}$$

Equation (8) provides a simple way to calculate the total activity coefficient from the activity coefficient *γ*_B_ of component B in a solution. Equation (8) can be further expressed as $\gamma =\sqrt[{n}]{\prod_{B}\gamma_{B}^{n_{B}}}$, where *n*_B_ is the number of moles of component B. *n* is the total number of moles, $n=\underset{B}{\sum }n_{B}$. In this sense, *γ* is a geometric mean of *γ*_B_. When *γ* > 1, the solution has a positive deviation from the ideal solution; when *γ* < 1, the solution has a negative deviation; when *γ* = 1, the solution is an ideal solution.

Gibbs free energy, *G*, can be expressed as $G=\underset{B}{\sum }n_{B}\mu_{B}$ [1]. Substituting the chemical potential *μ*_B_ in Equation (3) and *a*_B =_ *x*_B_*γ*_B_ into the expression of *G* gives

(9) $$G=G^{*}+RTln[\underset{B}{\prod }\left(\left(\gamma_{B}^{n_{B}}x_{B}^{n_{B}}\right)\right)]$$

where $G^{*}=\sum_{B}n_{B}\mu_{B}^{*}$. Since *γ^n^ =* $\underset{B}{\prod }\gamma_{B}^{n_{B}}$, it can be found easily that $\underset{B}{\prod }\left(\gamma_{B}^{n_{B}}x_{B}^{n_{B}}\right)$ = *γ^n^*$\underset{B}{\prod }\left(x_{B}^{n_{B}}\right)$. Like the total activity coefficient, the total concentration *x* can be defined similarly as *x^n^* ≡ $\underset{B}{\prod }x_{B}^{n_{B}}$. It can also be expressed further as $\ln x=\underset{B}{\sum }x_{B}\ln x_{B}$. The total activity *a* of a solution can then be defined as *a* ≡ *γx*. Thus, Equation (9) can be rewritten as

(10) $$G=G^{*}+nRT\ln a$$

In this way, the Gibbs free energy function has a very similar form as the chemical potential in Equation (3).

### 3.2. Proof of Intersection of the Curves of γ and All γ_B_ Versus x_B_ at the Stationary Point of the γ Curve

If the curves of *γ*_B_ versus *x*_B_ intersect at some point *x*_C_, *γ*_1_ = *γ*_2_ = …… = *γ*_n_ = *β*, where *β* is a parameter. Then Equation (8) becomes ln*γ* = $\ln\beta \underset{B}{\sum }x_{B}$. Since the sum of all *x*_B_ is 1, *γ* = *β*. This means that the curve of *γ* versus *x*_B_ intersects with all of the activity coefficient curves of *γ*_B_ versus *x*_B_ at the same point *x*_C_. Next, it will be proven that this intersection point is a stationary point of the curve of the total activity coefficient. The derivative of Equation (8) with respect to the molar concentration *x*_C_ at *γ* = *β* is

(11) $$\frac{1}{\gamma }{\left.\frac{\partial \gamma }{\partial x_{C}}\right|}_{\gamma =\beta }=\underset{B}{\sum }{x_{B}\left.\frac{\partial \ln\gamma_{B}}{\partial x_{C}}\right|}_{\gamma_{B}=\beta }+\ln\beta \frac{\partial }{\partial x_{C}}\underset{B}{\sum }x_{B}$$

Since $\underset{B}{\sum }x_{B}$ = 1, the second term on the right side of Equation (11) is equal to 0. Under isothermal and isobaric conditions, the Gibbs–Duhem equation, $\underset{B}{\sum }x_{B}d\mu_{B}$ = 0, becomes [1]

(12) $$\;\underset{B}{\sum }x_{B}d\ln\gamma_{B}+\underset{B}{\sum }x_{B}d\ln x_{B}=0$$

During the derivation of Equation (12), the expression of the chemical potential in Equation (3) is used. When it is divided by *dx*_C_, Equation (12) is changed to

(13) $$\underset{B}{\sum }x_{B}\frac{\partial \ln\gamma_{B}}{\partial x_{C}}+\underset{B}{\sum }\frac{\partial x_{B}}{\partial x_{C}}=0$$

The second term on the left side of Equation (13) is equal to 0 because $\underset{B}{\sum }x_{B}$ = 1. Therefore, the first term on the left side of Equation (13) is also equal to 0. Thus, the partial derivative ∂*γ*/∂*x*_C_ at *γ* = *β* in Equation (11) is equal to 0, which indicates that this point *x*_C_ is the stationary point of total activity coefficient curve. This is the end of proof.

### 3.3. Relationship Between the Total Activity Coefficient and the Excess Functions

The excess Gibbs energy *G^E^* of a mixture of liquids is defined as the difference between the Gibbs free energies of the actual solution and the hypothetical ideal solution [1]. According to reference [1], *G^E^* can be expressed as

(14) $$G^{E}=RT\underset{B}{\sum }n_{B}\ln\gamma_{B}$$

In comparison with the expression of the total activity coefficient *γ* in Equation (8), Equation (14) can be further written as

(15) $$G^{E}=nRT\ln\gamma$$

Equation (15) demonstrate the relationship between *G^E^* and *γ*. Both *G^E^* and *γ* can be exploited to show the deviation of actual solution from the hypothetical ideal solution. The excess volume *V^E^* is another excess property of a mixture of liquids. *V^E^* can be expressed as [1]

(16) $$V^{E}=\underset{B}{\sum }n_{B}(V_{B}-V_{B}^{*})$$

Comparison of Equation (16) with Equation (4) leads to

(17) $$V^{E}=nRT\underset{B}{\sum }x_{B}{\left(\frac{\partial \ln\gamma_{B}}{\partial P}\right)}_{T}$$

On the other hand, the partial derivative of the total activity coefficient in Equation (8) with respect to pressure is

(18) $$\frac{\partial \ln\gamma }{\partial P}=\underset{B}{\sum }x_{B}\frac{\partial \ln\gamma_{B}}{\partial P}+\underset{B}{\sum }\gamma_{B}\frac{\partial \ln x_{B}}{\partial P}$$

Substituting Equation (18) into Equation (17) gives

(19) $$V^{E}=nRT{\left(\frac{\partial \ln\gamma }{\partial P}\right)}_{T}-nRT\underset{B}{\sum }ln\gamma_{B}\frac{\partial x_{B}}{\partial P}$$

Equation (19) can be used to discuss the effect of pressure on *γ* once the excess volume is available. For a binary solution, Equation (19) can be rewritten as

(20) $${\left(\frac{\partial \ln\gamma }{\partial P}\right)}_{T}=\frac{V_{m}^{E}}{RT}+\frac{\partial x_{B}}{\partial P}ln\frac{\gamma_{B}}{\gamma_{A}}$$

where $V_{m}^{E}$ is the excess molar volume.

## 4. Conclusions

In this paper, the total activity coefficient is exploited to study the deviation of a solution from the hypothetical ideal solution. It is proven for a solution that the curve of total activity coefficient and all curves of activity coefficients of components will intersect at one point, which is a stationary point of the total activity coefficient curve. Two negative-deviation solutions and four positive-deviation solutions are investigated in this study. For the former, the intersection point is a minimum point of the concave curve of the total activity coefficient. As for the latter, the intersection point is a maximum point of the convex curve of the total activity coefficient. Like the total activity coefficient *γ*, the total activity *a* and total concentration *x* are also defined. The expression of Gibbs free energy in terms of *γ*, *a*, and *x* is thus very similar to the form of chemical potential.

Variations in the total activity coefficient with the molar concentration in negative and positive solutions studied here can be interpreted from the viewpoint of the intermolecular interaction. As for the negative (positive) deviation binary solutions, the magnitudes of the total activity coefficient at the minimum (maximum) point of the total activity coefficient curve becomes bigger as the intermolecular attraction of the solute and solvent become weaker. Furthermore, the minimum points of the total activity coefficient curves in the plots of *γ* versus the molar concentration of the negative solutions move horizontally to the side of the pure component with strong intermolecular attraction. In opposition to the negative-deviation solutions, the maximum points of the total activity coefficient curves in the plots of *γ* versus the molar concentration of the positive solutions moves horizontally to the side of the pure component with weak intermolecular attraction.

Finally, the effect of pressure on the total activity coefficient is investigated. For the negative solution of tetrahydrofuran + 1,1,2,2-tetrachloroethane, the total activity coefficient will decrease with the increase in pressure for smaller molar concentrations of tetrahydrofuran and increase with the pressure for relatively large molar concentrations of tetrahydrofuran. However, in opposition to the negative-deviation solution, the total activity coefficient of the positive-deviation solution of ethyl isobutyrate + 1-butanol increases with the pressure for small molar concentrations of ethyl isobutyrate at first and then decreases with the increase in pressure for big molar concentrations. For both negative- and positive-deviation solutions, the molar concentration of the dividing point is the same as the molar concentration of the intersection point of the curves of the total activity coefficient and the activity coefficient of the components.

## Data Availability

Data will be made available on request.

## Supplementary Materials

The following supporting information can be downloaded at: https://www.mdpi.com/article/10.3390/molecules30081681/s1, Table S1: The total activity coefficients *γ* of the negative-deviation systems CHCl_3_ + *c*-(CH_2_)_5_O and CHCl_3_ + *c*-(CH_2_)_5_NH at 333.15 K; Table S2: The total activity coefficients *γ* of the positive-deviation systems (CH_3_)_2_C=CH_2_ + CH_3_OH and (CH_3_)_2_C=CH_2_ + CH_3_CH_2_OH at 323.15 K; Table S3: The total activity coefficients *γ* of the positive-deviation systems (CH_3_)_2_C=CH_2_ + (CH_3_)_2_CHOH and (CH_3_)_2_C=CH_2_ + CH_3_CH_2_CH(OH)CH_3_ at 323.15 K.
