# Supplementary material for: Studies on the Deviation of a Solution from the Hypothetical Ideal Solution with the Total Activity Coefficient
Source: Molecules. 2025 Apr 9;30(8):1681. doi: 10.3390/molecules30081681 (PMC12029493; doi:10.3390/molecules30081681)
Supplement: Supplementary file 1 [file molecules-30-01681-s001.zip › molecules-3546976-supplementary.pdf]

Studies on deviation of a solution from the hypothetic ideal solution with the total activity coefficient

(Supplementary materials)

Ye-Qiu Zhou[a], Feiwu Chen[a]*, Yu Zhou[b]*

[a]*Department of Chemistry and Chemical Engineering, School of Chemistry and Biological Engineering, University of Science and Technology Beijing, Beijing 100083, China*

[b]*College of Chemistry and Chemical Engineering, Qingdao University, Qingdao 266071, China*

*** Corresponding author.**

E-mail address: chenfeiwu@ustb.edu.cn **(F. Chen).**

The following are included as supporting information for this paper:

Total 3 Pages, 3 Tables.

Table S1. The total activity coefficients γ of the negative deviation systems $CHCl_3$ + c-$(CH_2)_5O$ and $CHCl_3$ + c-$(CH_2)_5NH$ at 333.15 K.

$x(CHCl_3)$	$\gamma(CHCl_3 + c\text{-}(CH_2)_5O)$	$x(CHCl_3)$	$\gamma(CHCl_3 + c\text{-}(CH_2)_5NH)$
0.0000	1.0000	0.0000	1.0000
0.0298	0.9705	0.0680	0.9178
0.0736	0.9294	0.1242	0.8607
0.1363	0.8789	0.1896	0.8053
0.2150	0.8273	0.3223	0.7306
0.3149	0.7808	0.3872	0.7097
0.4242	0.7524	0.4545	0.7007
0.5308	0.7452	0.5160	0.6990
0.6244	0.7575	0.5754	0.7045
0.6825	0.7710	0.6412	0.7217
0.6903	0.7758	0.6831	0.7388
0.7101	0.7852	0.7519	0.7718
0.7631	0.8072	0.8673	0.8578
0.8453	0.8577	0.9424	0.9283
0.9458	0.9434	1.0000	1.0000
0.9767	0.9754		
1.0000	1.0000		

Table S2. The total activity coefficients γ of the positive deviation systems $(CH_3)_2C{=}CH_2$ + CH_3OH and $(CH_3)_2C{=}CH_2$ + CH_3CH_2OH at 323.15 K.

$x((CH_3)_2C{=}CH_2)$	$\gamma((CH_3)_2C{=}CH_2 + CH_3OH)$	$x((CH_3)_2C{=}CH_2)$	$\gamma((CH_3)_2C{=}CH_2 + CH_3CH_2OH)$
0.0000	1.0000	0.0000	1.0000
0.0309	1.0636	0.0353	1.0566
0.0503	1.1107	0.0538	1.0944
0.0971	1.1996	0.1002	1.1673
0.1452	1.2966	0.1480	1.2328
0.1956	1.3902	0.1974	1.3093
0.2469	1.4743	0.2474	1.3729
0.2981	1.5448	0.2983	1.4392
0.3493	1.6081	0.3488	1.4861
0.4005	1.6531	0.3998	1.5349
0.4024	1.6516	0.3984	1.5267
0.4515	1.6840	0.4474	1.5619
0.5008	1.6978	0.4971	1.5785
0.5501	1.7029	0.5468	1.5855
0.5995	1.6896	0.5972	1.5797
0.6488	1.6587	0.6475	1.5633
0.6982	1.6168	0.6979	1.5260
0.7485	1.5498	0.7484	1.4820
0.7975	1.4764	0.8006	1.4166
0.8473	1.3881	0.8516	1.3357
0.8967	1.2830	0.9035	1.2372
0.9468	1.1548	0.9531	1.1188
0.9853	1.0419	0.9738	1.0654
1.0000	1.0000	1.0000	1.0000

Table S3. The total activity coefficients γ of the positive deviation systems $(CH_3)_2C{=}CH_2$ + $(CH_3)_2CHOH$ and $(CH_3)_2C{=}CH_2$ + $CH_3CH_2CH(OH)CH_3$ at 323.15 K.

$x((CH_3)_2C{=}CH_2)$	$\gamma((CH_3)_2C{=}CH_2 + (CH_3)_2CHOH)$	$x((CH_3)_2C{=}CH_2)$	$\gamma((CH_3)_2C{=}CH_2 + CH_3CH_2CH(OH)CH_3)$
0.0000	1.0000	0.0000	1.0000
0.0414	1.0534	0.0402	1.0439
0.0653	1.0916	0.0578	1.0618
0.1065	1.1358	0.1049	1.1153
0.1531	1.1991	0.1529	1.1633
0.2015	1.2546	0.2013	1.2137
0.2500	1.2998	0.2507	1.2552
0.2993	1.3510	0.3000	1.2958
0.3490	1.3877	0.3507	1.3330
0.3983	1.4267	0.4016	1.3562
0.4491	1.4501	0.4512	1.3811
0.5092	1.4689	0.4877	1.3964
0.5502	1.4710	0.5013	1.3913
0.4493	1.4502	0.5259	1.3969
0.4983	1.4655	0.5475	1.4025
0.5964	1.4663	0.5970	1.3971
0.6458	1.4568	0.6465	1.3890
0.6954	1.4302	0.6961	1.3691
0.7461	1.3980	0.7455	1.3444
0.7943	1.3464	0.7966	1.2954
0.8441	1.2855	0.8466	1.2486
0.8945	1.2135	0.8978	1.1804
0.9447	1.1142	0.9524	1.0916
0.9862	1.0305	0.9925	1.0151
1.0000	1.0000	1.0000	1.0000
